# Record linkage study of the pathogen‐specific burden of respiratory viruses in children

**DOI:** 10.1111/irv.12508

**Published:** 2017-10-30

**Authors:** Faye J. Lim, Christopher C. Blyth, Parveen Fathima, Nicholas de Klerk, Hannah C. Moore

**Affiliations:** ^1^ Wesfarmers Centre of Vaccines and Infectious Diseases Telethon Kids Institute The University of Western Australia West Perth WA Australia; ^2^ School of Paediatrics and Child Health The University of Western Australia Perth WA Australia; ^3^ Department of Infectious Diseases Princess Margaret Hospital for Children Perth WA Australia; ^4^ PathWest Laboratory Medicine WA Princess Margaret Hospital for Children Perth WA Australia

**Keywords:** epidemiology, hospitalization, human parainfluenza virus, human respiratory syncytial virus, medical record linkage

## Abstract

**Background:**

Reliance on hospital discharge diagnosis codes alone will likely underestimate the burden of respiratory viruses.

**Objectives:**

To describe the epidemiology of respiratory viruses more accurately, we used record linkage to examine data relating to all children hospitalized in Western Australia between 2000 and 2012.

**Patients/Methods:**

We extracted hospital, infectious disease notification and laboratory data of a cohort of children born in Western Australia between 1996 and 2012. Laboratory records of respiratory specimens collected within 48 hours of admission were linked to hospitalization records. We calculated the frequency and rates of virus detection. To identify groups where under‐ascertainment for respiratory viruses was greatest, we used logistic regression to determine factors associated with failure to test.

**Results and conclusions:**

Nine percentage of 484 992 admissions linked to a laboratory record for respiratory virus testing. While 62% (n = 26 893) of laboratory‐confirmed admissions received respiratory infection diagnosis codes, 38% (n = 16 734) had other diagnoses, notably viral infection of unspecified sites. Of those tested, incidence rates were highest for respiratory syncytial virus (247 per 100 000 child‐years) followed by parainfluenza (63 per 100 000 child‐years). Admissions among older children and those without a respiratory diagnosis were associated with failure to test for respiratory viruses. Linked data can significantly enhance diagnostic codes when estimating the true burden of disease. In contrast to current emphasis on influenza, respiratory syncytial virus and parainfluenza were the most common viral pathogens among hospitalized children. By characterizing those failing to be tested, we can begin to quantify the under‐ascertainment of respiratory viruses.

## 1 INTRODUCTION

Acute lower respiratory infections (ALRI) include conditions such as bronchiolitis, pneumonia and influenza. The true burden of disease is difficult to calculate with estimates heavily influenced by identification methods. Globally, an estimated 10 per 1000 children aged less than 5 years were hospitalized for ALRI in 2010 in the developed world.^1^ Using linked administrative data, we estimated that in Western Australia (WA) the true rate was significantly higher; approximately 45 per 1000 children were hospitalized for ALRI before their second birthday, with a much higher burden among Aboriginal and Torres Strait Islander children (hereafter referred to as Aboriginal).^2^

Viruses associated with ALRI include influenza viruses, respiratory syncytial virus (RSV) and human metapneumovirus (hMPV). Current vaccines targeting ALRI viral pathogens are limited to influenza; however, multiple RSV vaccine candidates are in clinical trials.^3^ Accurate pathogen‐specific estimates are paramount before planning or evaluating prevention programmes.

At present, most retrospective studies using hospital admission data to investigate the aetiology of ALRI begin by selecting children with an International Classification of Disease (ICD) discharge code for ALRI. ICD codes alone are insufficient to provide accurate estimates of the true burden of respiratory pathogens because they are poorly sensitive,^4^ with limited use of pathogen‐specific codes. Furthermore, data are often from selected hospitals and may not be representative of the wider population. One method for more accurate calculations of the pathogen‐specific burden for a whole population is record linkage.

Record linkage combines data from multiple sources relating to the same person.^5^ Access to large, population‐level data sets at relatively low cost is one advantage of record linkage over prospective studies.^6^ We have shown that record linkage is a feasible and valid method for describing the pathogen‐specific aetiology of ALRI.^7^, ^8^ It is also useful for identifying conditions and populations where current databases underestimate the burden of disease.

We first sought to more accurately describe the pathogen‐specific burden of respiratory viruses (specifically by age‐specific rates and clinical diagnoses) identified in a cohort of WA‐born children hospitalized between 2000 and 2012. As significant portions of hospitalized children are not tested for respiratory viruses, their viral burden is probably underestimated. Therefore, we also aimed to characterize those who were not tested so that statistical modelling could be used to assign viruses to those who were not tested, based on demographic and admission factors.

## 2 PATIENTS AND METHODS

### 2.1 Setting and data sources

WA covers 2.5 million square kilometres with a population of approximately 2.5 million people at June 2015.^9^ Through the WA Data Linkage Branch, we identified a birth cohort of children born in WA between 1996 and 2012 from the Midwives Notifications System, Birth and Death Registries. We then extracted data relating to these children from the Hospital Morbidity Data Collection, PathWest Laboratory Medicine Database (PathWest) and the Western Australian Notifiable Infectious Diseases Database (WANIDD). All live births were included in the following analyses.

### 2.2 Hospital data

As PathWest data were only available from 2000, we restricted hospital records to those with an admission date between January 2000 and December 2012. Only hospital records with an admission and discharge date during the study period were included (hereafter referred to as hospital admissions). Admissions on or after the date of death were considered post‐mortem admissions and excluded.

Clinical diagnosis was classified using a hierarchy of ALRI, upper respiratory tract infections (URTI), or other, with priority given to ALRI. Admissions were classified as ALRI if they had a principal or codiagnosis ICD‐10‐AM (ICD 10th revision, Australian Modification) code for pneumonia, bronchiolitis, influenza, unspecified ALRI, bronchitis or whooping cough (codes listed in Table S1).^10^ Admissions with a principal or codiagnosis of URTI, such as otitis media and sinusitis, were classified as URTI (Table S1).^11^ All other admissions were classified as “other” and further subdivided based on principal diagnosis codes (Table S1).

Interhospital transfers were defined as multiple adjacent records of the same person where either (i) the admission date was identical to the discharge date of the prior record; or (ii) the admission date occurred before the discharge date of the prior record; or (iii) both admission and discharge dates fell within the time span of the prior record. These records were collapsed and considered a single admission. A child was coded as admitted to an intensive care unit (ICU) if they spent at least 1 day in ICU as recorded on the Hospital Morbidity Data Collection. Mechanical ventilation was defined as receipt of at least an hour of continuous ventilatory support or having procedure codes (classified using the 7th edition of the Australian Classification of Health Interventions) for airway management, invasive or non‐invasive ventilatory support (Table S1).

### 2.3 Laboratory data

PathWest is the sole public pathology provider in WA and services all but three hospitals admitting paediatric patients in the state. It also processes referred samples from private pathology laboratories in WA. Further details on PathWest are provided elsewhere.^7^ WANIDD is managed by the WA Department of Health and collects information on all notifiable infectious diseases in WA. We combined PathWest and WANIDD data on respiratory specimens collected from the birth cohort between January 2000 and December 2012.

We included laboratory data from nasal/nasopharyngeal (NP), throat, tracheal, bronchial, sputum, lung, pleural fluid, blood or serum specimens. Nasal/NP specimens included combined nose and throat, nasopharyngeal, per‐ and postnasal swabs and aspirates. Specimens where respiratory virus testing was requested and used one or more detection methods (including antigen detection [eg, immunofluorescent antibodies], polymerase chain reaction [PCR], viral culture and serology) were included. We focussed on respiratory viruses as parallel data on testing for bacterial pathogens were incomplete. Moreover, the sensitivity and specificity of current microbiology diagnostics for bacterial ALRI are relatively poor.

Respiratory viruses routinely tested on respiratory specimens included RSV, influenza A and B, human adenovirus and parainfluenza types 1‐3. Testing for hMPV was available from 2003 and routinely tested from 2008. Testing for other respiratory viruses, like human picornaviruses, was performed only on request. Picornaviruses were detected by PCR and viral culture but were not routinely speciated into rhinovirus or enterovirus. A specimen was deemed to test positive if one or more of adenovirus, influenza virus (any type), parainfluenza virus (any type), hMPV, picornavirus (unspeciated), rhinovirus or RSV were detected by antigen detection, PCR, culture or serology. Admissions where both picornavirus and rhinovirus were detected were coded as rhinovirus, whereas if only an unspecified picornavirus was identified, it was coded as a picornavirus admission.

Laboratory records were merged with a hospital record if respiratory specimens were collected 48 hours before or after the admission date. If the same person had multiple admissions for different reasons within 48 hours, laboratory records were linked to the ALRI admission. If the same person had multiple admissions for the same reason within 48 hours, laboratory records were linked to the admission closest to the date of specimen collection.

### 2.4 Statistical analysis

After merging hospital and laboratory records, we compared the clinical characteristics of admissions classified as ALRI, URTI and other. We then identified those with at least one virus detected and calculated the frequency and incidence of each virus by diagnosis, age and Aboriginal status. Incidence rates were calculated using person‐time‐at‐risk as the denominator, derived from dates of birth, death and end of the study (31 December 2012). Aboriginal children were identified using a derived variable provided by the WA Data Linkage Branch.^12^

We used logistic regression to identify admission‐specific factors associated with failing to have a virology test (outcome variable), clustering by person to allow for multiple admissions of the same person. Variables were selected based on clinical plausibility and/or a p‐value of less than 0.05 compared to the base model. These were included in the multivariable model to calculate adjusted odds ratios (aOR) and 95% confidence intervals (CI). All variables except admission year were included as categorical variables. Admission year was included as a continuous variable. All admissions were included in the adjusted models. The same model was used to calculate aOR of failing to test for a respiratory virus among those who received an ALRI diagnosis.

Data cleaning and descriptive analyses were performed in IBM SPSS (version 23). Regression models were developed in Stata Corp. STATA (version 14.1). Chi‐squared test for proportions, incidence rate ratios and exact 95% CI were calculated using EpiBasic (version 3).^13^ Ethical approvals were obtained from the WA Department of Health Human Research Ethics Committee, the WA Aboriginal Health Ethics Committee and the University of Western Australia Human Research Ethics Committee.

## 3 RESULTS

**Figure 1:**
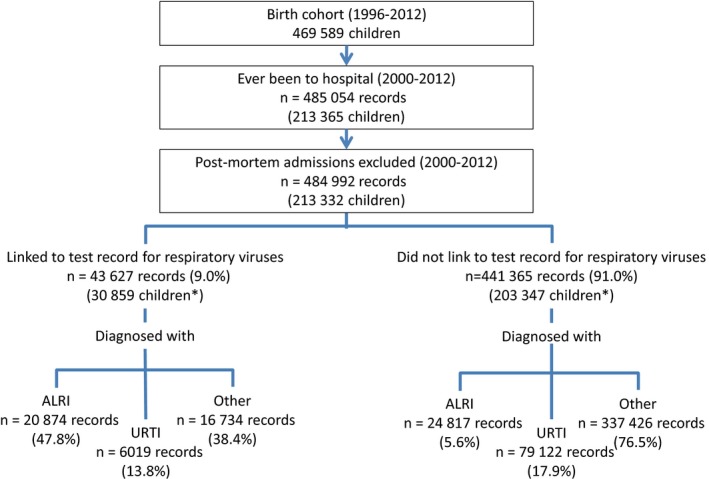
Flow chart of testing for respiratory viruses and diagnosis. Total number of children admitted in each category may not equal the total number of children hospitalized as a child may be hospitalized more than once

Of 469 589 children born between January 1996 and December 2012, 31 348 (6.7%) were Aboriginal and 240 237 (51.2%) were boys. Singleton births accounted for 97.0% of the cohort and 2428 children (0.5%) had died by 2012, resulting in 3 753 975.3 person‐years in total. Of those who died, ALRI was listed as the cause of death for 76 children (3.1%). Between January 2000 and December 2012, 45.4% (n = 213 365) of the cohort were hospitalized at least once, contributing to 485 054 admissions (Figure 1). Of these, 62 were considered post‐mortem admissions and excluded. Of the remaining admissions, 9.0% linked to a laboratory record for respiratory virus testing (Figure 1).

### 3.1 Comparing disease burden using hospital and laboratory data

We observed that 9.4% (n = 45 691) of all admissions had an ALRI diagnosis code (Figure 1), with bronchiolitis (n = 20 075, 43.9%) and pneumonia (n = 13 528, 29.6%) the most common diagnoses. However, only 45.7% (20 874/45 691) of admissions with an ALRI diagnosis linked to a laboratory record. Similarly, 17.6% (n = 85 141) of all admissions had a URTI diagnosis, but only 7.1% (6019/85 141) linked to a laboratory record (Figure 1).

Of all laboratory‐confirmed admissions, 47.8% had an ALRI diagnosis (n = 20 874; Figure 1). Over 70% of laboratory‐confirmed admissions with an ALRI diagnosis concerned children aged less than 2 years old at admission (Table S2). The majority of these children were diagnosed with bronchiolitis (n = 11 413) or pneumonia (n = 4484). Of the laboratory‐confirmed admissions with an URTI diagnosis, acute upper respiratory infection (n = 5558) was the most common diagnosis.

We noted that 38.4% of laboratory‐confirmed admissions did not have an ALRI or URTI diagnosis (Figure 1). These were commonly coded as viral infection of an unspecified site (n = 3338, 19.9%); other infections (n = 1786, 10.7%); asthma (n = 1595, 9.5%); or breathing abnormalities (n = 1228, 7.3%; Table 1). Approximately 12.5% of these admissions related to children aged 5 years or more at admission and 12.3% of these admissions included a stay in ICU (Table S2).

**Table 1:** Frequency and incidence rates of respiratory viruses per 100 000 child‐y among those who were tested

Description	Tested	Any virus		RSV		Parainfluenza		Influenza		Rhinovirus		Adenovirus		Unspeciated picornavirus^a^		hMPV	
	N	n	% tested	n	rate	n	rate	n	rate	n	rate	n	rate	n	rate	n	rate
ALRI diagnoses	20 874	12 192	58.4	7590	213.7	1201	33.8	1795	50.5	579	16.3	691	19.5	165	4.6	751	21.1
Acute bronchiolitis	11 413	7495	65.7	5959	167.8	540	15.2	102	2.9	320	9.0	408	11.5	62	1.7	435	12.2
Pneumonia	4484	1966	43.8	1067	30.0	235	6.6	265	7.5	146	4.1	153	4.3	41	1.2	190	5.4
Unspecified ALRI	2864	1036	36.2	474	13.3	209	5.9	61	1.7	87	2.4	98	2.8	53	1.5	108	3.0
Influenza due to identified virus	1611	1546	96.0	21	0.6	187	5.3	1357	38.2	<5	0.1	17	0.5	<5	0.1	16	0.5
Whooping cough	322	64	19.9	24	0.7	13	0.4	5	0.1	12	0.3	6	0.2	5	0.1	<5	0.0
Bronchitis	180	85	47.2	45	1.3	17	0.5	5	0.1	12	0.3	9	0.3	<5	0.0	<5	0.0
URTI	6019	1904	31.6	580	16.3	339	9.5	117	3.3	304	8.6	369	10.4	141	4.0	125	3.5
Other diagnoses	16 734	3651	21.8	598	16.8	680	19.1	248	7.0	681	19.2	441	12.4	946	26.6	179	5.0
Viral infection of unspecified site	3338	917	27.5	112	3.2	212	6.0	89	2.5	115	3.2	187	5.3	193	5.4	42	1.2
Other infections	1786	417	23.3	37	1.0	30	0.8	20	0.6	29	0.8	49	1.4	257	7.2	9	0.3
Asthma	1595	512	32.1	138	3.9	33	0.9	10	0.3	222	6.3	14	0.4	83	2.3	23	0.6
Cystic fibrosis	655	19	2.9	<5	0.1	9	0.3	<5	0.0	<5	0.1	<5	0.1	0	0.0	0	0.0
Other respiratory	1064	406	38.2	79	2.2	235	6.6	25	0.7	28	0.8	20	0.6	8	0.2	21	0.6
Breathing abnormalities	1228	272	22.1	87	2.4	25	0.7	<5	0.1	30	0.8	32	0.9	87	2.4	23	0.6
Fever	677	118	17.4	19	0.5	15	0.4	16	0.5	20	0.6	22	0.6	29	0.8	<5	0.0
Convulsions	459	57	12.4	11	0.3	11	0.3	10	0.3	5	0.1	13	0.4	7	0.2	<5	0.1
Abnormal clinical signs	657	65	9.9	11	0.3	10	0.3	9	0.3	15	0.4	11	0.3	11	0.3	<5	0.1
Other	5275	868	16.5	100	2.8	100	2.8	64	1.8	214	6.0	91	2.6	271	7.6	55	1.5
Total	43 627	17 747	40.7	8769	246.9	2220	62.5	2160	60.8	1564	44.0	1501	42.3	1252	35.3	1055	29.7

a Unspeciated picornavirus includes some detections of rhinovirus but these could not be distinguished from other types of picornaviruses (eg, enterovirus).

ALRI, acute lower respiratory infections, URTI, upper respiratory tract infection, RSV, respiratory syncytial virus, hMPV, human metapneumovirus. Rate presented is per 100 000 child‐y.

### 3.2 Pathogen‐specific burden of respiratory viruses

**Figure 2:**
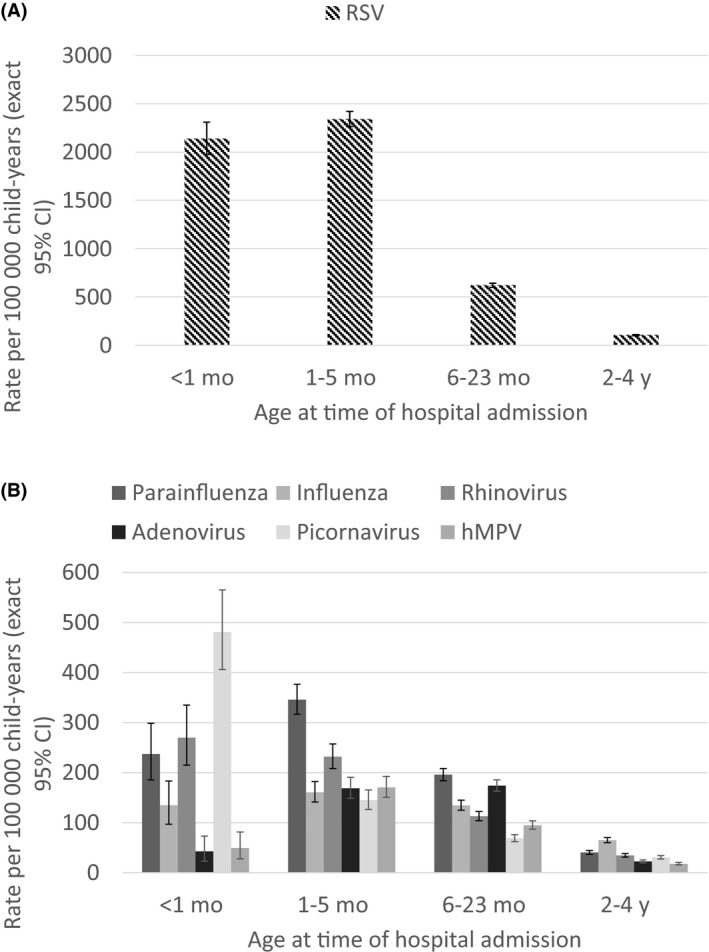
Incidence rates for (A) RSV and (B) other viruses among children aged <5 years. Note the differences in scale between A and B

Overall, 40.7% (n = 17 747) of laboratory‐confirmed admissions had at least one respiratory virus detected. Virus detection was highest among those diagnosed with ALRI (n = 12 192, 58.4%) and among children aged 6‐23 months at admission (n = 7087, 39.9%). Approximately 20.6% (n = 3651) of laboratory‐confirmed admissions with at least one virus detected did not have an ALRI or URTI diagnosis (Table 1). RSV was the most commonly detected virus, with an incidence rate that was four times higher (95% CI=3.8‐4.1) than the next most common virus (Table 1). RSV incidence rates were highest among children under 6 months of age (Figure 2A) and among those diagnosed with bronchiolitis (Table 1).

Unspeciated picornavirus, RSV, parainfluenza and rhinovirus had similar incidence rates among children with other diagnoses (Table 1). Rates of unspeciated picornavirus were highest among infants aged less than 1 month (Figure 2B). On the other hand, rates of parainfluenza and influenza virus peaked in children aged 1‐5 months. Among all laboratory‐confirmed admissions with parainfluenza detected, parainfluenza type three was the most common (n = 1548, 69.7%), followed by parainfluenza type one (n = 487, 21.9%). Influenza A (n = 1683, 76.7%), predominantly A/H1N1 (n = 327), was the most common type among those with influenza.

Viruses were detected more frequently in Aboriginal (n = 2931, 45.2% positive) compared to non‐Aboriginal children (n = 14 816, 39.9% positive; p < .001). However, these differences disappeared when further segregated by diagnoses. Virus distribution did not differ between Aboriginal and non‐Aboriginal children, although rates were at least twice as high in Aboriginal children for all viruses except for unspeciated picornavirus, which was 1.7 times higher (95% CI=1.4‐2.0) compared to non‐Aboriginal children. Two or more viruses were seen in approximately 4.1% (n = 723) of admissions testing positive for at least one virus, most commonly with RSV‐adenovirus (n = 206) and RSV‐parainfluenza pairs (n = 114).

### 3.3 Exploring under‐ascertainment of respiratory viruses

Univariable analyses suggested that age at admission, diagnosis, length of stay, hospital type and mechanical ventilation were strongly associated with failure to test for respiratory viruses (Table 2). These variables remained significant after adjusting for all other variables in the multivariable model.

**Table 2:** Logistic regression of admission‐specific factors associated with failing to test for respiratory viruses

Description	Total	Tested	Univariable		Multivariable	
	N	n	OR	(95% CI)	aOR	(95% CI)
Admission year^a^			1.03	(1.02‐1.03)	0.95	(0.94‐0.95)
Age at admission						
<1 mo	23 656	3271	Ref			
1‐5 mo	40 216	11 262	0.41	(0.39‐0.43)	0.72	(0.68‐0.76)
6‐23 mo	112 146	16 787	0.91	(0.87‐0.95)	1.11	(1.06‐1.17)
2‐4 y	146 021	8106	2.73	(2.60‐2.87)	2.32	(2.20‐2.45)
5‐9 y	113 179	3240	5.44	(5.08‐5.84)	4.31	(4.05‐4.58)
10‐16 y	49 774	961	8.15	(7.37‐9.01)	7.87	(7.25‐8.55)
Season of admission						
Summer (December–February)	104 327	6363	2.24	(2.17‐2.31)	1.51	(1.45‐1.56)
Autumn (March–May)	114 864	7812	1.99	(1.94‐2.05)	1.44	(1.39‐1.49)
Winter (June–August)	133 383	16 940	Ref			
Spring (September–November)	132 418	12 512	1.39	(1.36‐1.43)	1.06	(1.03‐1.10)
Diagnosis						
ALRI	45 691	20 874	Ref			
URTI	85 141	6019	11.06	(10.67‐11.46)	4.71	(4.54‐4.90)
Other	354 160	16 734	16.96	(16.49‐17.45)	14.68	(14.21‐15.15)
ICU admission						
No	465 854	40 100	4.10	(3.92‐4.29)	1.12	(1.06‐1.20)
Yes	19 138	3527	Ref			
Length of stay						
1 d	204 348	2971	Ref			
2 d	140 618	10 095	0.19	(0.18‐0.20)	0.29	(0.28‐0.30)
3 or more days	140 026	30 561	0.05	(0.05‐0.06)	0.11	(0.11‐0.12)
Inter‐hospital transfers						
No	475 260	42 358	Ref			
Yes	9732	1269	0.65	(0.61‐0.69)	1.27	(1.18‐1.37)
Hospital type						
Tertiary	214 629	30 030	Ref			
Metropolitan (public)	44 930	4197	1.58	(1.52‐1.64)	3.32	(3.18‐3.47)
Rural (public and private)	102 880	5739	2.75	(2.65‐2.86)	9.14	(8.80‐9.49)
Metropolitan (private)	122 553	3661	5.28	(5.07‐5.50)	5.63	(5.40‐5.87)
Mechanical ventilation						
No	482 140	42 279	9.32	(8.60‐10.11)	1.88	(1.70‐2.09)
Yes	2852	1348	Ref			

a Included as a continuous variable.

ALRI, acute lower respiratory infections; URTI, upper respiratory infections; OR, odds ratio; aOR, adjusted odds ratio; ICU, intensive care unit. Multivariable model adjusted for all other variables listed.

Children over 5 years of age and those without an ALRI diagnosis had at least fourfold greater odds of not being tested (Table 2). Furthermore, admissions to non‐tertiary hospitals had at least threefold odds of not being tested than admissions to tertiary hospitals. When restricted to admissions with an ALRI diagnosis, results were similar for all variables except ICU admission and interhospital transfers (Table S3). Children admitted to regular wards with an ALRI diagnosis had 0.71 times the odds of failing to test for respiratory viruses compared to those admitted to ICU (Table S3). Likewise, admissions following interhospital transfers had lower odds of not receiving a test than those who were transferred (Table S3).

## 4 DISCUSSION

As ICD codes alone can underestimate the burden of respiratory viruses, we used linked laboratory and hospital data to determine more accurate estimates of the virus‐specific burden of ALRI among hospitalized children. We determined that the overall incidence rate of RSV was 247 per 100 000 child‐years, with a similar rate among those diagnosed with ALRI. Incidence rates for other respiratory viruses ranged from 30 to 63 per 100 000 child‐years. A third of all laboratory‐confirmed admissions were not diagnosed with ALRI or URTI. Children over 5 years of age, not receiving a diagnosis of ALRI and admission to a non‐tertiary hospital, were strongly associated with failing to test for respiratory viruses.

RSV, parainfluenza and influenza were the most commonly detected viruses, with the greatest burden among children under 6 months old. Much of previous preventative effort towards viral ALRI has focused on influenza. More recently, investigations have turned towards RSV and parainfluenza, with 60 RSV vaccine candidates currently in clinical or preclinical development^3^ and a dozen parainfluenza vaccine candidates.^14^, ^15^ Given the relative greater burden of RSV and parainfluenza, increased efforts are needed to address these two pathogens. Prevention of all three viruses would greatly reduce the burden of ALRI and potentially 20%‐30% of other hospitalizations as well.

Approximately 38% of all laboratory‐confirmed admissions were not coded as ALRI or URTI, with 22% of these admissions testing positive for at least one respiratory virus. These do not appear to be solely attributable to asymptomatic virus identification as many of these viruses were infrequently detected in asymptomatic children.^16^ Moreover, these laboratory‐confirmed admissions were mostly unspecified diagnosis codes (eg, viral infections of unspecified site) or non‐specific clinical symptoms (eg, breathing abnormalities). This highlights the limitations of using ICD codes alone when calculating disease estimates, as documented elsewhere.^4^, ^17^, ^18^ Previous studies also support the notion that coding algorithms, particularly for less well‐defined diseases, are needed for accurate disease estimates.^19^ These are crucial to policy development and are often used as baseline data when evaluating the impact of said policies. Our findings further highlight the need to look beyond ICD codes when estimating infectious disease burden and the impact of vaccination programs.

While there were many laboratory‐confirmed admissions without a respiratory code, over half of all ALRI admissions were not tested for respiratory viruses, implying that the burden of specific viruses is still underestimated. Without the capacity to directly influence testing patterns and because it is economically unfeasible to impose universal testing, one possibility to address this issue is by statistical modelling. By characterizing those testing positive for particular viruses, we could extrapolate virus “detection” to those with similar characteristics but not tested. This method was used on similar data in England,^20^ and a future study using these data is planned.

We noted that admissions to private metropolitan hospitals had five times greater odds of failing to test compared to tertiary hospitals. As a limited number of private hospitals do not routinely use PathWest laboratories, it is difficult to differentiate testing practices from specimen referral pathways. Despite this, the frequency of virus detection in private metropolitan hospitals in this data set was comparable to tertiary hospitals, as was the distribution of viruses (data not shown). Inclusion of laboratory data from private pathology providers would thus further enhance our disease estimates.

Previous work validating linked laboratory data in WA suggested that approximately 75% of ALRI‐related admissions should have an associated laboratory record for respiratory pathogen testing.^8^ Notwithstanding the exclusion of bacterial pathogens and commonly associated tests (eg, blood culture), the proportion of laboratory‐confirmed admissions with an ALRI diagnosis is still suboptimal despite improving our extraction protocols. While linkage errors (eg, misidentification of individuals across data sets) may have partially contributed to this issue, it is likely to be minimal as approximately 95% of PathWest records were linked successfully (personal communication, WA Data Linkage Branch).

We were unable to investigate the role of comorbidities (eg, immunosuppression) and antiviral use on testing practices and the frequency of virus detection. We also could not examine the emerging role of rhinovirus in severe respiratory infections^21^ as picornaviruses were not routinely speciated. While we focussed on respiratory viruses, competition and interactions with bacterial pathogens are likely to influence incidence rates of individual viruses investigated here. Inclusion of data on bacterial pathogens is the next step in assessing the burden of all respiratory pathogens.

## 5 CONCLUSION

Despite these limitations, this is one of the few studies, to our knowledge, to quantify the population‐level burden of respiratory viruses in children, irrespective of diagnosis. It is also the first to use individual, rather than aggregated, person‐time‐at‐risk data to more accurately estimate the rate of respiratory virus infection. This has enabled reporting of pathogen‐specific incidence rates of these viruses, the majority of which are not notifiable. Access to data on all admissions for a whole population, in addition to both positive and negative test results, is a major strength of this study. Using these data, we have shown that respiratory viruses are pervasive and their prevention could reduce the burden of respiratory and non‐respiratory hospitalizations. These data provide a framework for further, in‐depth studies to enhance current and future preventative strategies. We plan to use these data to investigate temporal trends and risk factors for specific viruses and their combinations in the context of changes in testing patterns.

## CONFLICTS OF INTEREST

The authors have no conflicts of interest relevant to this article to disclose.

## AUTHORS CONTRIBUTION

Drs Moore and Blyth conceptualized and designed the study, guided data cleaning and analyses, reviewed and revised the manuscript and approved the final manuscript as submitted. Professor de Klerk conceptualized and designed the study, guided data analyses, reviewed and revised the manuscript and approved the final manuscript as submitted. Ms Lim cleaned and analysed the data, drafted the initial manuscript, reviewed and revised the manuscript and approved the final manuscript as submitted. Dr Fathima cleaned the data, reviewed and revised the manuscript and approved the final manuscript as submitted.
