# Supplementary material for: Record linkage study of the pathogen‐specific burden of respiratory viruses in children
Source: Influenza Other Respir Viruses. 2017 Oct 30;11(6):502–10. doi: 10.1111/irv.12508 (PMC5705691; doi:10.1111/irv.12508)
Supplement: Supplementary file 1 [file IRV-11-502-s001.docx]

# Table S1 – Diagnosis and procedure codes used

| **Description** | | **Codes used** | **Type** |
| --- | --- | --- | --- |
| **ALRI** | |  |  |
|  | Whooping cough | A37 | Diagnosis |
|  | Pneumonia | J12-18 | Diagnosis |
|  | Acute bronchiolitis | J21 | Diagnosis |
|  | Influenza due to identified virus | J09-J10 | Diagnosis |
|  | Unspecified ALRI | J22 | Diagnosis |
|  | Bronchitis | J20, J40 | Diagnosis |
| **URTI** | |  |  |
|  | Diphtheria | A36.0 – A36.2 | Diagnosis |
|  | Otitis media and other ear conditions | B05.3, H65-H67, H68.0, H72, H73.0, H83.0, H92.1 | Diagnosis |
|  | Mastoiditis and related conditions | H70, H75.0 | Diagnosis |
|  | Acute upper respiratory infections (incl. pharyngitis) | J00-J04, J06 | Diagnosis |
|  | Epiglottitis | J05.1 | Diagnosis |
|  | Chronic sinusitis | J32 | Diagnosis |
|  | Other nose, tonsil or adenoid disorders | J34.0, J35.0, J36 | Diagnosis |
| **Other diagnosis** | |  |  |
|  | Viral infection of unspecified site | B34 | Diagnosis |
|  | Other infections | AXX, BXX unless included in other categories | Diagnosis |
|  | Asthma | J45, J46 | Diagnosis |
|  | Cystic fibrosis | E84 | Diagnosis |
|  | Other respiratory diseases | JXX unless included in other categories | Diagnosis |
|  | Breathing abnormalities (incl. cough) | R05, R06 | Diagnosis |
|  | Convulsions (incl. febrile) | R56, P90 | Diagnosis |
|  | Fever | R50 | Diagnosis |
|  | Abnormal clinical signs, symptoms and laboratory findings | RXX unless included in other categories | Diagnosis |
| **Mechanical ventilation** | |  |  |
|  | Airway management | 22007-00, 22007-01, 22008-00, 22008-01, 90179-02, 92035-00, 92041-00 | Procedures |
|  | Non-invasive ventilatory support | 92209-XX | Procedures |
|  | Ventilatory support | 13882-XX | Procedures |

Note: “-XX” denotes all sub-codes within a particular block. Diagnosis codes were based on the International Statistical Classification of Diseases and Related Health Problems codes, 10th revision, Australian Modification. Both principal and co-diagnosis fields were used to classify admissions. Procedure codes were based on the Australian Classification of Health Interventions, 7th edition. ALRI=acute lower respiratory infections, URTI=upper respiratory tract infections.
