# Supplementary material for: Record linkage study of the pathogen‐specific burden of respiratory viruses in children
Source: Influenza Other Respir Viruses. 2017 Oct 30;11(6):502–10. doi: 10.1111/irv.12508 (PMC5705691; doi:10.1111/irv.12508)
Supplement: Supplementary file 2 [file IRV-11-502-s002.docx]

# Table S2 – Characteristics of hospital admissions among those who were tested for respiratory viruses by diagnosis

| **Description** | | **ALRI (n=20,874)** | | **URTI (n=6019)** | | **Other reason (n=16,734)^a^** | |
| --- | --- | --- | --- | --- | --- | --- | --- |
|  |  | **n** | **%** | **n** | **%** | **n** | **%** |
| Age at admission | |  |  |  |  |  |  |
|  | <1 month | 962 | 4.6 | 363 | 6.0 | 1946 | 11.6 |
|  | 1-5 months | 6479 | 31.0 | 1084 | 18.0 | 3699 | 22.1 |
|  | 6-23 months | 8781 | 42.1 | 2573 | 42.7 | 5433 | 32.5 |
|  | 2-4 years | 3110 | 14.9 | 1434 | 23.8 | 3562 | 21.3 |
|  | 5-9 years | 1192 | 5.7 | 462 | 7.7 | 1586 | 9.5 |
|  | 10-16 years | 350 | 1.7 | 103 | 1.7 | 508 | 3.0 |
| Season of admission | |  |  |  |  |  |  |
|  | Summer (Dec-Feb) | 2103 | 10.1 | 980 | 16.3 | 3280 | 19.6 |
|  | Autumn (Mar-May) | 2923 | 14.0 | 1217 | 20.2 | 3672 | 21.9 |
|  | Winter (Jun-Aug) | 9969 | 47.8 | 2095 | 34.8 | 4876 | 29.1 |
|  | Spring (Sep-Nov) | 5879 | 28.2 | 1727 | 28.7 | 4906 | 29.3 |
| Hospital type | |  |  |  |  |  |  |
|  | Tertiary (public) | 12,836 | 61.5 | 4098 | 68.1 | 13,096 | 78.3 |
|  | Metropolitan (public) | 2637 | 12.6 | 625 | 10.4 | 935 | 5.6 |
|  | Rural (public or private) | 3605 | 17.3 | 706 | 11.7 | 1428 | 8.5 |
|  | Metropolitan (private) | 1796 | 8.6 | 590 | 9.8 | 1275 | 7.6 |
| Admitted to ICU | | 1168 | 5.6 | 306 | 5.1 | 2053 | 12.3 |
| Required mechanical ventilation | | 632 | 3.0 | 93 | 1.5 | 623 | 3.7 |

Note: ALRI=acute lower respiratory infections, URTI=upper respiratory tract infections. Percentages may not equal 100 due to missing data and rounding.

^a^ Admissions in this group were commonly coded as unspecified viral or other infections (n=3338); other infections (n=1786); asthma (n=1595); breathing abnormalities (n=1228) or other respiratory diseases (n=1064). Refer to Table 1 for a more detailed breakdown of this group.
