# Supplementary material for: Record linkage study of the pathogen‐specific burden of respiratory viruses in children
Source: Influenza Other Respir Viruses. 2017 Oct 30;11(6):502–10. doi: 10.1111/irv.12508 (PMC5705691; doi:10.1111/irv.12508)
Supplement: Supplementary file 3 [file IRV-11-502-s003.docx]

# Table S3 - Logistic regression of admission-specific factors associated with failing to test for respiratory viruses among those with an ALRI diagnosis

| **Description** | | **Total** | **Tested** | **Univariable** | | **Multivariable** | |
| --- | --- | --- | --- | --- | --- | --- | --- |
|  |  | **N** | **N** | **OR** | **(95% CI)** | **aOR** | **(95% CI)** |
| Admission year^a^ | |  |  | 0.97 | (0.96-0.97) | 0.93 | (0.92-0.94) |
| Age at admission | |  |  |  |  |  |  |
|  | <1 month | 1316 | 962 | Ref |  |  |  |
|  | 1-5 months | 10,735 | 6479 | 1.79 | (1.57-2.03) | 1.39 | (1.19-1.62) |
|  | 6-23 months | 19,423 | 8781 | 3.29 | (2.91-3.73) | 2.36 | (2.03-2.73) |
|  | 2-4 years | 9377 | 3110 | 5.48 | (4.81-6.24) | 4.81 | (4.12-5.62) |
|  | 5-9 years | 3798 | 1192 | 5.94 | (5.10-6.92) | 6.87 | (5.77-8.17) |
|  | 10-16 years | 1042 | 350 | 5.37 | (4.40-6.56) | 7.89 | (6.12-10.16) |
| Season of admission | |  |  |  |  |  |  |
|  | Summer (Dec-Feb) | 5879 | 2103 | 1.89 | (1.78-2.01) | 1.48 | (1.37-1.60) |
|  | Autumn (Mar-May) | 7753 | 2923 | 1.74 | (1.65-1.84) | 1.45 | (1.36-1.55) |
|  | Winter (Jun-Aug) | 19,444 | 9969 | Ref |  |  |  |
|  | Spring (Sep-Nov) | 12,615 | 5879 | 1.21 | (1.15-1.26) | 1.04 | (0.99-1.10) |
| ICU admission | |  |  |  |  |  |  |
|  | No | 44,219 | 19,733 | 4.28 | (3.76-4.86) | 0.71 | (0.59-0.85) |
|  | Yes | 1472 | 1141 | Ref |  |  |  |
| Length of stay | |  |  |  |  |  |  |
|  | 1 day | 2468 | 702 | Ref |  |  |  |
|  | 2 days | 12,698 | 4267 | 0.79 | (0.71-0.86) | 0.55 | (0.48-0.62) |
|  | 3 or more days | 30,525 | 15,905 | 0.37 | (0.33-0.40) | 0.25 | (0.23-0.29) |
| Inter-hospital transfers | |  |  |  |  |  |  |
|  | No | 44,633 | 20,240 | Ref |  |  |  |
|  | Yes | 1058 | 634 | 0.55 | (0.49-0.63) | 0.67 | (0.57-0.80) |
| Hospital type | |  |  |  |  |  |  |
|  | Tertiary | 16,654 | 12,836 | Ref |  |  |  |
|  | Metropolitan (public) | 5411 | 2637 | 3.54 | (3.31-3.78) | 4.37 | (4.07-4.69) |
|  | Rural (public and private) | 18,135 | 3605 | 13.55 | (12.80-14.34) | 16.13 | (15.18-17.14) |
|  | Metropolitan (private) | 5491 | 1796 | 6.92 | (6.45-7.42) | 8.52 | (7.88-9.22) |
| Mechanical ventilation | |  |  |  |  |  |  |
|  | No | 44,882 | 20,242 | 4.35 | (3.68-5.13) | 1.30 | (1.02-1.66) |
|  | Yes | 809 | 632 | Ref |  |  |  |

Note: ALRI=acute lower respiratory infections, OR=odds ratio, aOR=adjusted odds ratio. Multivariable model adjusted for all other variables listed and clustered by individuals.

^a^ Included as a continuous variable.
